# Performance Enhancement of Ti/IrO_2_-Ta_2_O_5_ Anode through Introduction of Tantalum–Titanium Interlayer via Double-Glow Plasma Surface Alloying Technology

**DOI:** 10.3390/nano14141219

**Published:** 2024-07-18

**Authors:** Mingshuai Guo, Yueren Liu, Yonglei Xin, Likun Xu, Lili Xue, Tigang Duan, Rongrong Zhao, Junji Xuan, Li Li

**Affiliations:** 1 College of Materials Science and Chemical Engineering, Harbin Engineering University, Harbin 150001, China; gms281980310@163.com (M.G.);; 2 National Key Laboratory of Marine Corrosion and Protection, Luoyang Ship Material Research Institute, Qingdao 266237, China; 3 Luoyang Ship Material Research Institute, Luoyang 471023, China

**Keywords:** oxide anode, iridium oxide, tantalum oxide, tantalum–titanium interlayer, double-glow plasma surface alloying technology

## Abstract

Ti/IrO_2_-Ta_2_O_5_ electrodes are extensively utilized in the electrochemical industries such as copper foil production, cathodic protection, and wastewater treatment. However, their performance degrades rapidly under high current densities and severe oxygen evolution conditions. To address this issue, we have developed a composite anode of Ti/Ta-Ti/IrO_2_-Ta_2_O_5_ with a Ta-Ti alloy interlayer deposited on a Ti substrate by double-glow plasma surface alloying, and the IrO_2_-Ta_2_O_5_ surface coating prepared by the traditional thermal decomposition method. This investigation indicates that the electrode with Ta-Ti alloy interlayer reduces the agglomerates of precipitated IrO_2_ nanoparticles and refines the grain size of IrO_2_, thereby increasing the number of active sites and enhancing the electrocatalytic activity. Accelerated lifetime tests demonstrate that the Ti/Ta-Ti/IrO_2_-Ta_2_O_5_ electrode exhibits a much higher stability than the Ti/IrO_2_-Ta_2_O_5_ electrode. The significant improvement in electrochemical stability is attributed to the Ta-Ti interlayer, which offers high corrosion resistance and effective protection for the titanium substrate.

## 1. Introduction

Anodes with mixed metal oxides coated on Ti substrate have been widely used in electrochemical industries, such as hydrometallurgy, copper foil production, electrodeposition, cathodic protection, and wastewater treatment [1,2,3,4,5,6]. The anodes are required to possess not only a large electrochemical activity but also a high stability for long-term operation. Among the various oxide anode materials working in oxygen-evolving conditions, the Ti/IrO_2_-Ta_2_O_5_ electrode with a composition of Ir: Ta = 70:30 in mole ratio is regarded as a premier choice due to its excellent performance, which has been testified in many applications [6,7,8]. However, the electrode is often subject to premature failure in harsh conditions, for example, when operating at a very large current density in an acidic environment [9,10,11,12,13,14]. The deactivation of the Ti/IrO_2_-Ta_2_O_5_ electrode is mainly related to the consumption of active oxides, the corrosion of the Ti substrate, and the formation of an insulating TiO_2_ layer between the active oxide coating and the substrate with electrolyte and evolved oxygen penetrating through the cracks [15,16].

To further improve the performance of Ti/IrO_2_-Ta_2_O_5_ electrodes, it is essential to enhance the corrosion resistance of the Ti substrate and suppress the generation of a non-conductive TiO_2_ film [17,18,19]. Thus, it is reasonable and effective to have a protective interlayer on the Ti substrate. The interlayer should have good conductivity and enough adhesion with the substrate and the top oxide coating [19,20,21]. Numerous interlayers, such as noble metal [22], no-noble metal [11,23,24], metallic oxide [25,26], and other conductive materials [18,20,27], have been extensively studied to prolong the service life of oxide-coated Ti electrodes. The non-precious metal interlayers are beneficial for industrial applications considering the advantage of balance between cost and performance. Tantalum is an excellent choice for the interlayer because of its high corrosion resistance and good conductivity [28]. In addition, the oxides generated in the sintering process are easily combined with IrO_2_-Ta_2_O_5_ coating to improve adhesion [13,29]. According to the study by Vercesi et al., the tantalum-based IrO_2_-Ta_2_O_5_ electrode had a much longer service life than the titanium-based electrode [29]. However, the high price of tantalum hinders the use of bulk metal as the substrate, and a Ta interlayer on the Ti substrate should be a reasonable way to tackle this problem. There have been many methods to prepare the Ta or Ta suboxide interlayer on Ti substrate, such as cold gas spray [24], plasma immersion ion implantation and deposition [30], thermal decomposition method in an inert atmosphere [31], and electrodeposition in a molten salt bath [28]. It has been demonstrated that the introduction of a Ta or Ta suboxide interlayer can definitely improve the performance of the Ti/IrO_2_-Ta_2_O_5_ electrode [24,28,31]. 

Double-glow plasma surface alloying technology (DGPSAT) is a surface modification process that diffuses metal elements into conductive materials to realize surface alloying [32,33,34,35]. The DGPSAT device mainly consists of an anode, source electrode (target material), cathode (workpiece), vacuum system, argon supply, cathode–anode DC power supply, and source–anode DC power supply [32]. In the case of a vacuum environment with two DC power supplies turned on, two glow discharges occur between the anode and cathode and the anode and source electrode. One glow discharge heats the cathode to the specific temperature to receive the transmitted atoms, while the second glow discharge bombards the source electrode. The atoms splashed from the source electrode diffuse to the cathode surface to form a surface alloy layer. In the DGPSAT process, atoms reach the substrate surface via thermal movement. A high vacancy concentration gradient is generated at a certain depth below the substrate surface under the bombardment of high-energy ions. The high vacancy concentration gradient reduces the diffusion activation energy of metal atoms, causing the diffusion rate in DGPSAT to be much higher than that of the ordinary metal diffusion process. 

Some efforts have been made to manufacture Ta coatings via DGPSAT to enhance corrosion resistance [33,36]. Specifically, Song et al. [33] demonstrated that the Ta coating deposited on a γ-TiAl surface using DGPSAT significantly improved oxidation resistance by preventing oxygen atoms from penetrating the substrate at high temperatures. Wei et al. [36] also found that a Ta coating deposited on Ti using the DGPSAT method obviously improved the corrosion resistance of the sample. The corrosion rate of the tantalum-modified layer in 10% and 40% sulfuric acid solution was only 30% and 1.2% of that of pure titanium, respectively. However, to the best of our knowledge, the use of DGPSAT to prepare a Ta alloy interlayer on a titanium substrate for the modification of a Ti/IrO_2_-Ta_2_O_5_ electrode has never been reported. In this paper, a composite electrode based on Ti was prepared with the Ta-Ti alloy interlayer formed via DGPSAT and the IrO_2_-Ta_2_O_5_ surface coating using the traditional thermal decomposition method. The microstructures of the Ta-Ti alloy interlayer and the Ti/Ta-Ti/IrO_2_-Ta_2_O_5_ composite anode were characterized, and the electrochemical performance and stability of the composite anode were studied. It is verified that the introduction of a Ta-Ti alloy interlayer via DGPSAT can significantly enhance the electrocatalytic activity and service life of Ti/IrO_2_-Ta_2_O_5_ electrode.

## 2. Experimental 

### 2.1. Preparation of Interlayer

A titanium plate (20 mm × 10 mm × 1.5 mm) of Grade TA2 (Baoji Titanium Industry Co., Ltd., Baoji, Shanxi, China) was used as the substrate. It was de-greased in 1 mol·L^−1^ NaOH solution at 90 °C for 1 h and etched in 10 wt% oxalic acid solution at 80 °C for 2 h. The etched titanium plate and a high-purity tantalum (99.99 wt%) disc (Ningxia Orient Tantalum Industry Co., Ltd., Shizuishan, Ningxia, China) with a size of Φ100 mm × 5 mm were used as the cathode and source electrode, respectively. The preparation of the Ta-Ti alloy coating was carried out in a double-glow plasma surface alloying device (Shenyang Scientific Instrument Development Center of China Academy of Sciences, Shenyang, China) [33,34], and the operation parameters are listed in Table 1. It has been demonstrated that depositing Ta using double-glow plasma surface alloying under these parameters is feasible [33,37]. The turning on of the two DC power supplies caused an unequal hollow cathode effect due to the difference in potential between the titanium substrate and the tantalum source. Due to the lower negative bias of the titanium substrate, Ta atoms sputtered from the tantalum source electrode were deposited on it after heating to 900 °C. Subsequently, a Ta-Ti alloy was formed via diffusion in depth under the action of thermodynamic and kinetic driving forces. All the chemicals and reagents used in this work were purchased from China Sinopharm Chemical Reagent Co, Ltd., Shanghai, China.

### 2.2. Preparation of Electrode

The IrO_2_-Ta_2_O_5_ coatings on different substrates were fabricated using the traditional thermal decomposition method [38]. H_2_IrCl_6_ and TaCl_5_ dissolved in n-butanol and hydrochloric acid with a nominal Ir:Ta = 70:30 mole ratio were used to prepare the precursor solution with a metallic concentration of 0.3 mol·L^−1^. The precursor solution was painted on the pretreated Ti substrate with or without the interlayer using a brush. The samples were dried for 10 min at 120 °C and then sintered for 15 min at 500 °C in a muffle furnace (OTF-1200X, Hefei Kejing Material Technology Co. Ltd., Hefei, China). These parameters of thermal decomposition processes have been widely adopted for the preparation of Ti/IrO_2_-Ta_2_O_5_ electrodes [6,38]. To achieve an oxide loading of about 7.0 g/m^2^, the brushing, drying, and sintering steps were repeated five times. Finally, the samples were annealed at 500 °C for 1 h. 

### 2.3. Surface Analysis and Electrochemical Measurement

The morphologies and chemical compositions of the Ta-Ti alloy interlayer and the oxide electrodes were detected using a scanning electron microscope (SEM, ULTRA 55, Carl Zeiss AG, Oberkochen, Germany) equipped with an EDS analyzer (Inca X-Max, Oxford Instruments, Abingdon, UK). The crystal structures of the interlayer and the electrodes were obtained via an X-ray diffractometer (XRD, Rigaku D/Max 2500, Tokyo, Japan) with Cu Kα radiation at 40 kV and 150 mA. The surfaces of the interlayer and the oxide coating were tested using an X-ray photoelectron spectrometer (XPS, Thermo ESCALAB 250Xi, Waltham, MA, USA) with a monochromatic Al Kα X-ray source (1486.6 eV).

The electrode samples used in this study all had the dimensions of 20 mm × 10 mm × 1.5 mm. Each electrochemical test used three replicated specimens to ensure accuracy. A multichannel electrochemical workstation (VMP3, Bio-logic, Claix, France) was used to measure the electrochemical properties of a sample in a three-electrode system with platinum as the counter electrode, the IrO_2_-Ta_2_O_5_ electrode as the working electrode, and the saturated calomel electrode as the reference electrode. All of the electrochemical tests except for those specified were carried out under ambient conditions in 0.5 mol·L^−1^ sulfuric acid solution. The linear sweep voltammetry (LSV) curves were recorded with a scan rate of 0.33 mV s^−1^. Cyclic voltammetry (CV) measurements were conducted at varying scan rates ranging from 10 mV s^−1^ to 100 mV s^−1^ within the potential range from 0.6 V to 0.8 V (vs. SCE). Electrochemical impedance spectroscopy (EIS) tests were performed at a potential of 1.4 V (vs. SCE) in a frequency range of 0.01 Hz–100 kHz with a signal amplitude of 5 mV. The accelerated life test of the electrode was carried out at an anodic current density of 3 A·cm^−2^ in 1 mol·L^−1^ H_2_SO_4_ solution at 40 °C charged by a direct current power (DC, DH1718E-4, Donghua Testing Technology Co., Ltd., Beijing, China). During the experiment, the temporal evolution of the cell voltage between the anode and cathode was monitored and recorded. The potentiodynamic polarization measurements for the Ta-Ti interlayer and Ti substrate were conducted in 1 mol·L^−1^ sulfuric acid at a scanning rate of 0.3 mV s^−1^.

## 3. Results and Discussion

### 3.1. Microstructure

**Figure 1:**
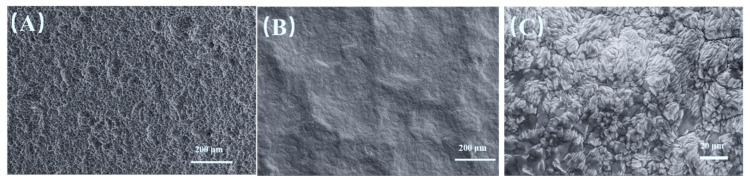
SEM morphologies of (**A**) Ti substrate after etching in hot oxalic acid, (**B**,**C**) Ta-Ti alloy interlayer deposited via DGPSAT. (**C**) is the enlarged photo of (**B**).

The surface morphologies of different samples, including the Ti substrate and the Ta-Ti alloy interlayer, are shown in Figure 1. The surface of an acid-etched Ti substrate (Figure 1A) presents a lot of small corrosion pits [39], which benefits the retention of precursor solution on the substrate surface [40], as well as the bonding of coating with the substrate. After the DGPSAT treatment, a coating (Ta-Ti interlayer) was deposited on the etched titanium substrate, as shown in Figure 1B. The etched titanium substrate presents a rough surface morphology, while the deposited coating shows a relatively flat and uniform morphology. The magnified SEM picture (Figure 1C) displays that the interlayer is composed of grains with agglomerated cellular particles in sizes of 0.2~1.0 μm inside. This change in surface morphology with the interlayer deposited would affect the microstructures of the top IrO_2_-Ta_2_O_5_ coatings.

EDS analysis demonstrated that the surface composition of the interlayer consisted mainly of tantalum (Ta), oxygen (O), and titanium (Ti) (Table 2). The small amount of O element should have derived from the slight oxidation of the surface. It can be found that a large amount of Ta, accounting for 92.83 atom %, and a little amount of Ti were observed on the surface of the interlayer. Ta and Ti elements should come from the Ta-Ti alloy interlayer, which has a thickness much larger than that detected using EDS.

**Figure 2:**
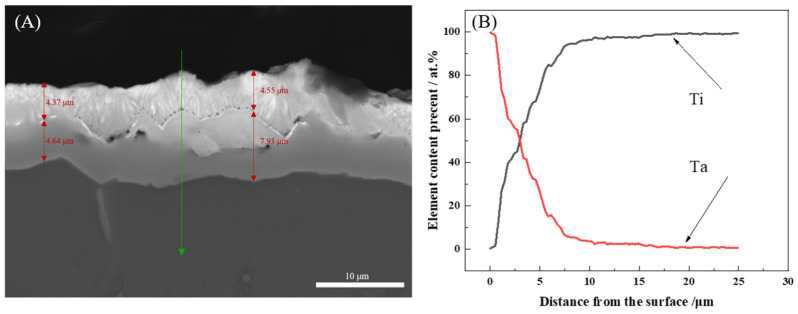
Cross-sectional morphology of Ta-Ti interlayer (**A**) and elements distribution with depth of Ta-Ti interlayer (**B**). The green line with arrow in (**A**) represents the location for EDS analysis by line scanning.

The cross-sectional morphology of the Ta-Ti alloy interlayer is shown in Figure 2A, which displays the microstructure of the interlayer changing with depth and can be divided into two stratums. The first stratum is on the top with a thickness of about 4 μm. The second stratum is light gray with a thickness of about 4–8 μm. The gray region below the interlayer is the Ti substrate. The prepared interlayer has a thickness of about 8–12 μm. EDS analysis with line scanning, as shown in Figure 2B, indicated that the Ta content decreased while the Ti content continuously increased with the depth, demonstrating that the interlayer is the Ta-Ti binary alloy with the composition changing along the depth. The first stratum has a content of Ta greater than 41% and almost 100% at the outermost superficial layer. The tantalum content in the second stratum is less than 41% and decreases with depth, indicating that Ti is rich in this stratum. Therefore, the Ta-Ti alloy interlayer prepared via DGPSAT has a gradient composition. Some micro-voids can be found at the interface between the original surface of the substrate and the interlayer. The surface of the etched Ti substrate is rough, which can lead to an inhomogeneous higher vacancy concentration gradient with the substrate bombarded by ions. This mechanism resulted in an uneven distribution of tantalum atom deposition from thermal movement, producing micro-voids of different sizes. Considering that the interlayer undergoes metallurgical bonding with the substrate via surface alloying, and the anode bears little load at working, some discrete micro-voids in the interlayer should not lead to the detachment of the coating. Although these micro defects can be tolerated in the composite anode, the quality of the deposited interlayer should be further improved later by the optimization of the substrate pretreatment and processing parameters.

**Figure 3:**
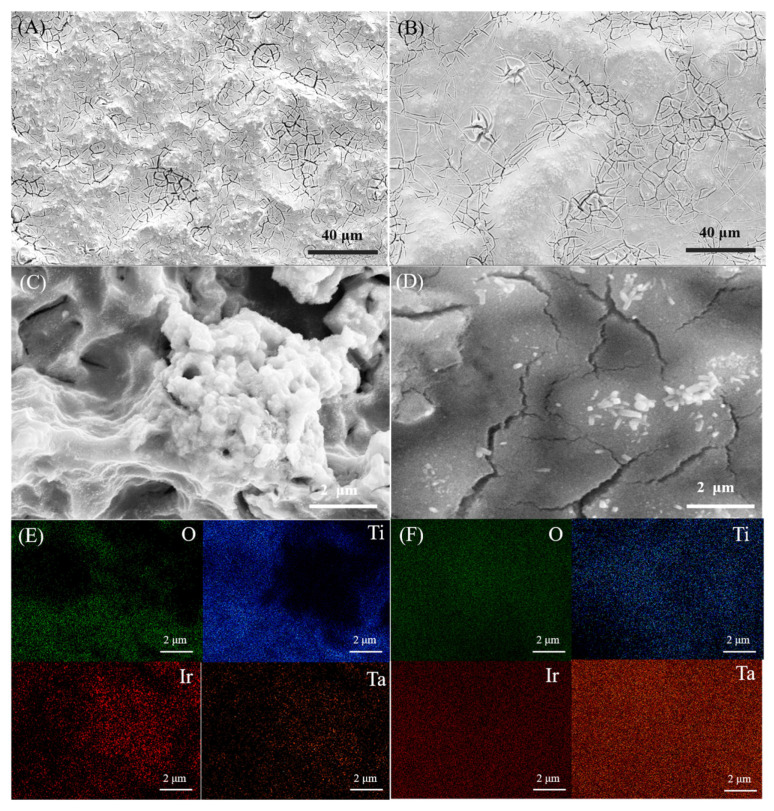
SEM morphologies (**A**–**D**) and EDS mapping (**E**,**F**) of Ti/IrO_2_-Ta_2_O_5_ anode (**A**,**C**,**E**) and Ti/Ta-Ti/IrO_2_-Ta_2_O_5_ composite anode (**B**,**D**,**F**). (**E**,**F**) are EDS mappings of (**C**,**D**), respectively.

Figure 3A,B show the SEM morphologies of Ti/IrO_2_-Ta_2_O_5_ and Ti/Ta-Ti/IrO_2_-Ta_2_O_5_ anodes. The surfaces of Ti/IrO_2_-Ta_2_O_5_ and Ti/Ta-Ti/IrO_2_-Ta_2_O_5_ anodes present a similar appearance with some “dried mud” cracks, which are very typical for metal oxide-coated titanium electrodes prepared using the thermal decomposition method [6,38]. The micro-cracks should be caused by the thermal stress produced during the preparation process due to the different thermal expansion coefficients between the substrate and the coating [31,41]. The surface of the Ti/IrO_2_-Ta_2_O_5_ electrode is more uneven than that of the Ti/Ta-Ti/IrO_2_-Ta_2_O_5_ electrode, which can be attributed to the different roughness of the etched Ti substrate and the Ta-Ti interlayer. There are more micro-cracks on the Ti/IrO_2_-Ta_2_O_5_ electrode surface than that on the Ti/Ta-Ti/IrO_2_-Ta_2_O_5_ electrode because the thick coating located at the deep pits or dimples on the substrate suffers higher thermal stress and is liable to cracking. The cracks can increase the area of contact between surface active sites and electrolytes, increasing electrochemical surface area, but they also accelerate the deactivation of the anode, with the substrate being attacked more easily [15]. 

Furthermore, in the magnified SEM images (Figure 3C,D), some agglomerates of white nano-scale particles are observed with precipitation on the electrode surfaces. Combined with EDS mapping analysis (Figure 3E,F), these white nano-scale particles were identified mainly as IrO_2_. It has been found that the agglomerates with nanoparticles determined as being IrO_2_ crystallites are often present on IrO_2_-Ta_2_O_5_ electrodes [15,42]. From Figure 3E, it is evident that IrO_2_ aggregation occurs, whereas Figure 3F shows a more uniform distribution of IrO_2_. These differences in surface morphology and EDS mapping suggest that adding the Ta-Ti interlayer reduces the agglomeration of IrO_2_ nanoparticles, which can enhance the electrocatalytic activity of an electrode due to a more homogeneous distribution of active sites. The precipitation of agglomerates should be related to the roughness of the substrate and the distribution of the thickness of the IrO_2_-Ta_2_O_5_ coating. The locally thin coating at the ridges on the etched Ti substrate facilitates the precipitation and aggregation of nano-IrO_2_ crystallites during thermal decomposition processes [31,38], while the introduction of the interlayer can lower the surface roughness, thus reducing the aggregation of nano-IrO_2_ particles. 

The results of EDS analysis on the oxide anodes are also listed in Table 2, which shows that Ir, Ta, Ti, O, and Cl elements are detected on the surfaces of Ti/Ta-Ti/IrO_2_-Ta_2_O_5_ and Ti/IrO_2_-Ta_2_O_5_ electrodes. A little chlorine existing in the oxide coating of the electrodes comes from the residual chloride precursor, which may not completely transform to oxides during sintering. The atomic ratio of Ir and Ta for Ti/IrO_2_-Ta_2_O_5_ electrode is about 69:31, close to the nominal composition of the IrO_2_-Ta_2_O_5_ coating (Table 2). The more Ti content detected for Ti/IrO_2_-Ta_2_O_5_ electrode implies that the oxide coating is thin and that X-rays can penetrate the coating or go through the micro-cracks to the Ti substrate. The Ti/Ta-Ti/IrO_2_-Ta_2_O_5_ electrode has a lower Ti content and a higher Ta content than the Ti/IrO_2_-Ta_2_O_5_ electrode relating to the thin oxide coating and the fact that the composition analyzed via EDS is a mixture of the oxide coating and the Ta-Ti interlayer. 

**Figure 4:**
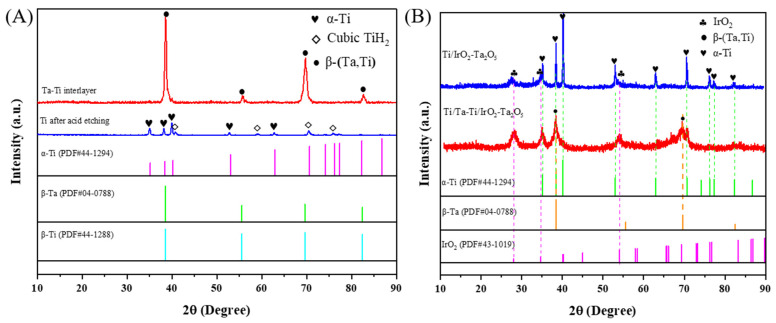
XRD patterns of Ta-Ti interlayer (**A**) and Ti/IrO_2_-Ta_2_O_5_ and Ti/Ta-Ti/IrO_2_-Ta_2_O_5_ electrodes (**B**).

The XRD patterns of the different samples are shown in Figure 4. The XRD pattern of acid-etched Ti displays characteristic peaks attributed to the cubic TiH_2_ phase, as well as the α-Ti phase. These results are in agreement with previous investigations [39]. The detection range in depth when using the XRD technique is generally limited to 10–30 μm, and the EDS analysis of the cross-sectional sample for the Ta-Ti interlayer revealed the coexistence of binary Ti and Ta elements at this depth. The diffraction peaks observed at 38.5°, 55.5°, 69.6°, and 82.5° for the Ta-Ti interlayer correspond to the β-Ta and β-Ti phases, indicating their simultaneous presence in the sample. It should be noted that the DGPSAT process was carried out at a temperature of approximately 900℃, which is adequate to form β-Ta and β-Ti [43,44,45]. Furthermore, based on the Ti-Ta binary phase diagram [46] and combined with the EDS analysis results, it can be found that the first stratum of the alloy interlayer is a solid solution of β-(Ta,Ti), having the same phase structure of β-Ta. For the second stratum, the Ti content is dominant, and the phase composition changes to β-(Ti,Ta). A similar phase structure was reported in the literature that the Ti-Ta alloys with a content of 20–50 atom % Ta produced via powder metallurgy after sintering all have a structure of β phase because Ti and Ta can form a continuous solid solution [47]. It was also found that the stable β-Ta can be formed in the Ta-Ti alloy when the Ta content reaches a certain value [36]. Whether it is β-Ti or β-Ta, their corrosion resistance is superior to that of α-Ti [29,48], indicating that the Ta-Ti interlayer can enhance the corrosion resistance of the substrate. 

The peaks of the Ti/IrO_2_-Ta_2_O_5_ and Ti/Ta-Ti/IrO_2_-Ta_2_O_5_ electrodes (Figure 4B) at 28.0°, 34.7°, and 53.5° can be attributed to the IrO_2_ rutile phase. There exist diffraction peaks of α-Ti substrate in the XRD pattern of the Ti/IrO_2_-Ta_2_O_5_ electrode, while β-(Ta,Ti) peaks appear for the composite electrode with the interlayer. No Ta_2_O_5_ peak was detected in both oxide electrodes, suggesting the phase is amorphous because the crystallization of Ta_2_O_5_ cannot occur at the low sintering temperature [39]. According to Scherrer’s formula [39,49], the average grain size of the active component of IrO_2_ can be calculated from the diffraction peaks of IrO_2_ for the oxide electrodes, which is reduced from about 12 nm to about 9 nm after adding the interlayer, indicating that the IrO_2_ nano-crystallites in the oxide coating are refined when the interlayer is applied. The reduction in grain size can produce a larger electrochemical active area, which is conducive to improving electrocatalytic activity [31].

**Figure 5:**
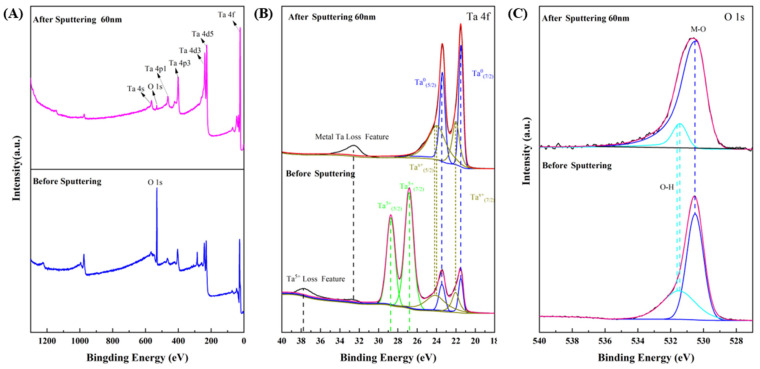
XPS spectra of Ta-Ti interlayer before and after sputtering to a depth of 60 nm. (**A**) full spectrum, (**B**) Ta-4f and (**C**) O-1s.

X-ray photoelectron spectroscopy (XPS) can be used to analyze the chemical state of the sample surface. The XPS full spectra of the top-surface and the sub-surface at 60 nm depth after ion sputtering of the Ta-Ti interlayer are shown in Figure 5A. The detection depth of XPS is generally less than 10 nm. As the full spectrum displays, the superficial surface of the interlayer was found to contain tantalum (Ta) and oxygen (O) elements, with no evidence of the presence of titanium (Ti). This observation suggests that the surface composition of the interlayer comprises metallic tantalum and tantalum oxide. 

Figure 5B,C show the XPS spectra of high-resolution electron binding energy for Ta 4f and O 1s. The Ta 4f peaks at around 21.5/23.5 eV and 26.8/28.75 eV are assigned to metallic Ta and Ta_2_O_5_ [50], respectively. While the peaks at 22/24.05 eV are attributed to low-valent tantalum oxide (Ta^x+^, 0 < x < 5) [50]. The absence of tantalum pentoxide on the sub-surface suggests that the oxidation of tantalum is limited to its top-surface during the DGPSAT process. A similar phenomenon of oxidation at the superficial surface was also observed in other high-vacuum preparation techniques relating to tantalum coatings, such as magnetron sputtering [51,52] and chemical vapor deposition [53], which can be explained by the high sensitivity of tantalum to oxygen at high temperatures [29]. The disappearance of the diffraction peak of tantalum pentoxide and the reduction of 0.1 eV in the binding energy of the lower-valence oxide were observed in the sub-surface, which can be attributed to the reduction of the oxide due to ion sputtering. The O 1s peaks at 530.55 eV are attributed to the lattice oxygen [54], and the peaks at 531.45 eV are assigned to hydroxides [55]. The stability of lattice oxygen is unaffected by ion bombardment, whereas the binding energy of hydroxides shifted 0.2 eV towards a positive direction, suggesting that they are predominantly located on the top-surface [42]. The element contents detected using XPS are listed in Table 3. It can be found that the oxygen content decreases significantly in the interlayer after ion sputtering to remove the superficial layer. The results indicate that the surface layer of the Ta-Ti interlayer is composed of metallic tantalum and conductive tantalum suboxide, and only the top-surface is oxidized to pentoxide.

**Figure 6:**
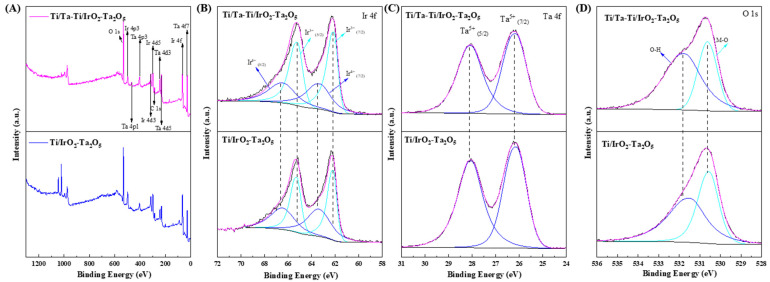
XPS spectra of Ti/IrO_2_-Ta_2_O_5_ and Ti/Ta-Ti/IrO_2_-Ta_2_O_5_ electrodes. (**A**) full spectrum, (**B**) Ir-4f, (**C**) Ta-4f, and (**D**) O-1s.

Figure 6 shows the XPS spectra of Ti/IrO_2_-Ta_2_O_5_ and Ti/Ta-Ti/IrO_2_-Ta_2_O_5_ electrodes. The full spectra of the oxide electrodes (Figure 6A) demonstrate that the oxide coatings are composed of Ir, Ta, and O. In the Ir 4f spectra, the binding energies located at 62.1/65.35 eV and 63.45/66.5 eV correspond to Ir^3+^ and Ir^4+^ species, respectively [56,57]. The coexistence of Ir^3+^ and Ir^4+^ species is often found in IrO_2_-Ta_2_O_5_ coating prepared via the thermal decomposition method [51,52,53], and the transformation of Ir^3+^/Ir^4+^ redox can facilitate the oxygen evolution reaction (OER), resulting in a reduction in the OER potential [58]. The binding energies at 26.1 and 28.15 eV in the Ta 4f spectrum correspond to Ta_2_O_5_. The O 1s spectrum presents two peaks at 530.65 eV and 531.6 eV belonging to the lattice oxygen and hydroxides, respectively. Therefore, the IrO_2_-Ta_2_O_5_ coatings prepared on either the Ta-Ti interlayer or the Ti substrate via the thermal decomposition method have almost no difference in electronic structure. For the Ti/IrO_2_-Ta_2_O_5_ electrode, the Ir/Ta ratio is observed to be a little large as compared to that for the Ti/Ta-Ti/IrO_2_-Ta_2_O_5_ electrode (see Table 3). This discrepancy can potentially be explained by the diffusion of tantalum from the interlayer [59]. Moreover, the Ir/Ta ratio for both electrodes is smaller than the nominal composition as well as the results when using EDS analysis (see Table 2), which can be attributed to the surface segregation phenomenon, leading to a lower concentration of Ir [60].

### 3.2. Electrocatalytic Activity 

**Figure 7:**
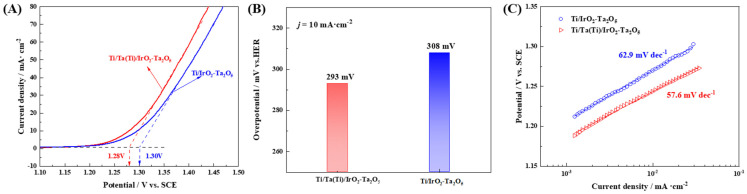
(**A**) Linear sweep voltammetry curves of Ti/IrO_2_-Ta_2_O_5_ and Ti/Ta-Ti/IrO_2_-Ta_2_O_5_ electrodes measured in 0.5 mol·L^−1^ H_2_SO_4_ solution. (**B**) The overpotential for oxygen evolution of different electrodes at the current density of 10 mA·cm^−2^. (**C**) Tafel slopes of different electrodes.

The electrochemical performance of the Ti/IrO_2_-Ta_2_O_5_ and Ti/Ta-Ti/IrO_2_-Ta_2_O_5_ electrodes was characterized using LSV, EIS and CV measurements. The LSV curves corrected with iR drop are shown in Figure 7A. It can be found that the Ti/Ta-Ti/IrO_2_-Ta_2_O_5_ electrode presents a lower OER onset potential and a larger current density for oxygen evolution at the constant working potential than the Ti/IrO_2_-Ta_2_O_5_ electrode. The Ti/Ta-Ti/IrO_2_-Ta_2_O_5_ electrode exhibits excellent OER activity, with an overpotential of 293 mV to reach the current density of 10 mA·cm^−2^ (Figure 7B). In contrast, the Ti/IrO_2_-Ta_2_O_5_ electrode requires a higher overpotential of 308 mV at the same current density. In addition, the Ti/Ta-Ti/IrO_2_-Ta_2_O_5_ electrode has a lower Tafel slope than the Ti/IrO_2_-Ta_2_O_5_ electrode for OER (Figure 7C). The LSV results indicate that the Ti/Ta-Ti/IrO_2_-Ta_2_O_5_ electrode exhibits a higher OER electrocatalytic activity than the Ti/IrO_2_-Ta_2_O_5_ electrode.

**Figure 8:**
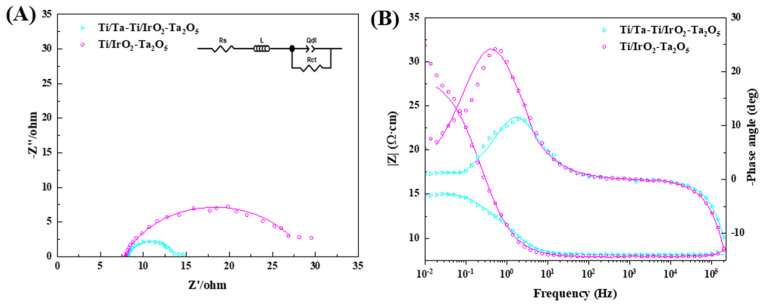
EIS spectra of Ti/IrO_2_-Ta_2_O_5_ and Ti/Ta-Ti/IrO_2_-Ta_2_O_5_ electrodes measured at 1.4 V vs. SCE in 0.5 mol·L^−1^ H_2_SO_4_ solution. (**A**) Nyquist plot and (**B**) Bode plot.

The EIS spectra recorded at 1.4 V (vs. SCE) in 0.5 mol·L^−1^ H_2_SO_4_ solution are shown in Figure 8A. For both electrodes, the Nyquist diagrams displayed a depressed semi-circle, indicating the presence of a relaxation process with a single time constant. However, the absence of a capacitive arc at higher frequencies is noteworthy as it suggests that neither electrode exhibited a reduction in the conductivity of its oxide coating or an insulating oxide layer formed on the substrate beneath the IrO_2_-Ta_2_O_5_ coating [6,15]. No semi-circle has been found in the high-frequency region, which proves that neither Ti substrate nor Ta-Ti interlayer has been further oxidized during the preparation of the top oxide coating via thermal decomposition. The small flat tails appearing at the low frequencies should be related to the bubble effect due to strong gas evolution [31]. The semi-circle diameter of the Ti/Ta-Ti/IrO_2_-Ta_2_O_5_ electrode is smaller than that of the Ti/IrO_2_-Ta_2_O_5_ electrode, indicating that the introduction of the Ta-Ti interlayer via DGPSAT can promote the electrocatalytic activity of the IrO_2_-Ta_2_O_5_ electrode. The equivalent circuit model in Figure 8A was used to fit the EIS spectra, and the corresponding parameters are given in Table 4. In the equivalent circuit, *R*_s_, *L*, *R*_ct_, and *Q*_dl_ are the solution resistance, the inductance, the charge transfer resistance, and the constant phase element, respectively. *Q*_dl_ is used to replace the double-layer capacitance to obtain a better simulation due to the dispersive effect from the rough surface of the electrode [31]. The inductance present at high frequency should be related to the interference in the electrical circuit. The *R*_ct_ of the Ti/Ta-Ti/IrO_2_-Ta_2_O_5_ electrode is smaller than that of the Ti/IrO_2_-Ta_2_O_5_ electrode, indicating that the Ta-Ti interlayer can enhance the electrocatalytic activity of oxygen evolution reaction. In addition, the Ti/Ta-Ti/IrO_2_-Ta_2_O_5_ electrode has a larger *Q*_dl_ than the Ti/IrO_2_-Ta_2_O_5_ electrode, suggesting that the electrode with the Ta-Ti interlayer has a larger electrochemical active surface area, which agrees with the LSV results.

**Figure 9:**
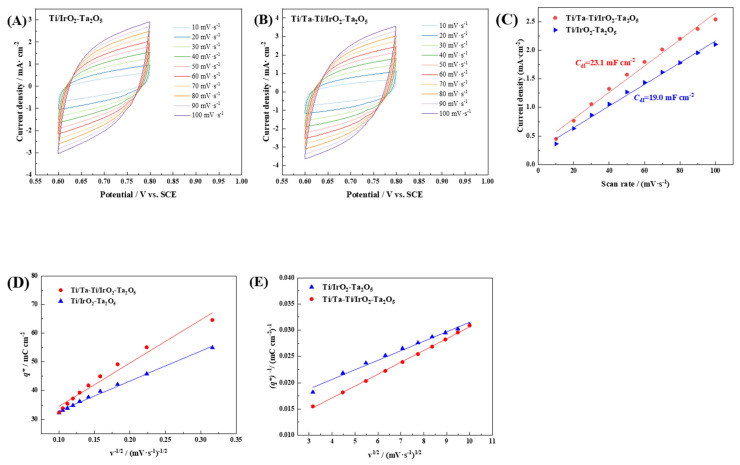
(**A**,**B**) Cyclic voltammograms between 0.6 V and 0.8 V at different sweep rates for Ti/IrO_2_-Ta_2_O_5_ and Ti/Ta-Ti/IrO_2_-Ta_2_O_5_ electrodes, respectively. (**C**) Evolution of current density at 0.7 V vs. scan rate for different electrodes. (**D**) The relationship between voltammetric charge (*q*^∗^) and the inverse square root of scanning rate (v^−1/2^) for different electrodes. (**E**) The relationship between the reciprocal voltammetric charge (*q*^∗^)^−1^ and square root of scanning rate (v^1/2^) for different electrodes.

The voltametric charge and electrochemical double-layer capacitance of the electrodes can be measured via cyclic voltammetry. The CV curves taken at different scan rates in the potential range of 0.6 V~0.8 V (vs. SCE) are given in Figure 9A,B. The relationship between the current density Δj (j_a_ − j_c_) at 0.7 V (vs. SCE) and the scanning rate is shown in Figure 9C, from which the double-layer capacitance can be derived as the slope of the fitting line. The *C*_dl_ value of the Ti/Ta-Ti/IrO_2_-Ta_2_O_5_ electrode (23.1 ± 0.9 mF·cm^−2^) is greater than that of the Ti/IrO_2_-Ta_2_O_5_ electrode (19.0 ± 0.5 mF·cm^−2^), as shown in Table 5. Given that there exists a correlation between the double-layer capacitance and the surface area of the electrode that participates in electrochemical reactions, which is commonly referred to as the electrochemically active surface area (EASA), it can be considered that an increase in *C*_dl_ will result in a corresponding increase in EASA [61]. The *C*_dl_ result demonstrates that the Ti/Ta-Ti/IrO_2_-Ta_2_O_5_ electrode has a larger EASA, thereby a higher electrocatalytic activity than the Ti/IrO_2_-Ta_2_O_5_ electrode. 

The voltammetric charge, *q*^∗^, obtained from the cyclic voltammetry curve is proportional to the number of electroactive sites present on the electrode surface [56]. At high scan rates, electrochemical reactions between active sites and electrolyte solutions occur more easily in the “outer” surface region. At low scan rates, the exchange reaction occurs predominantly in the “total” region (both the “outer” and “inner” active surface regions) [62]. The functional relationship between the voltammetric charge and the scanning rate can be described using the following equations [42]. The charge of the “outer” active surface in contact with the electrolyte, $q_{out}^{*}$, can be obtained from Equation (1). The “total” voltammetric charge, $q_{tot}^{*}$, can be obtained from Equation (2). The charge of the “inner” active surface hidden in pores and cracks, $q_{in}^{*}$, can be calculated from Equation (3).

(1) $$q^{*}=q_{out}^{*}+k_{1}v^{-0.5}$$

(2) $$q^{*-1}=q_{tot}^{*-1}+k_{2}v^{0.5}$$

(3) $$q_{tot}^{*}=q_{out}^{*}+q_{in}^{*}\;$$

where *k*_1_ and *k*_2_ are constants, *v* is the potential scanning rate. Figure 9D,E show the plots of the relationship between *q*^∗^ and *v* for Ti/IrO_2_-Ta_2_O_5_ and Ti/Ta-Ti/IrO_2_-Ta_2_O_5_ electrodes, and the derived voltammetric charges are also listed in Table 5. The total voltammetric charge observed for the Ti/Ta-Ti/IrO_2_-Ta_2_O_5_ electrode is significantly greater than that of the Ti/IrO_2_-Ta_2_O_5_ electrode, suggesting that the former possesses a larger electrochemically active surface area. The charge of the inner surface for both electrodes is much larger than that of the outer surface. It can also be found that the *q*^∗^_in_ for the Ti/Ta-Ti/IrO_2_-Ta_2_O_5_ electrode is much larger than that for Ti/IrO_2_-Ta_2_O_5_ electrode. These results demonstrate that the enhanced electrocatalytic activity of the Ti/Ta-Ti/IrO_2_-Ta_2_O_5_ electrode mainly originates from the large inside of EASA. Although the Ti/Ta-Ti/IrO_2_-Ta_2_O_5_ electrode surface has fewer micro-cracks, the dispersed nano IrO_2_ particles and the refinement of IrO_2_ crystallites with the interlayer added increase the number of electrocatalytic active sites, thereby enhancing the voltammetric charges [63]. 

### 3.3. Stability

**Figure 10:**
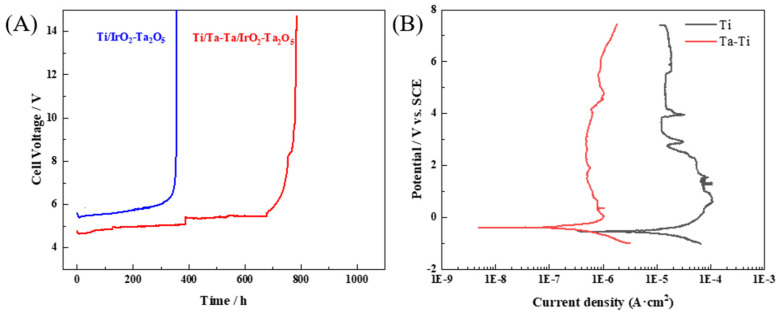
(**A**) Variation of cell voltage of Ti/IrO_2_-Ta_2_O_5_ and Ti/Ta-Ti/IrO_2_-Ta_2_O_5_ electrodes with time for the accelerated life testing at 3 A·cm^−2^ in 1 mol·L^−1^ H_2_SO_4_ solution. (**B**) Potentiodynamic polarization curves of Ta-Ti interlayer and Ti substrate in 1 mol·L^−1^ H_2_SO_4_ solution.

Accelerated life testing (ALT) is a commonly used method to assess the stability of Ti/IrO_2_-Ta_2_O_5_ anodes [8]. The ALT was carried out at 3 A·cm^−2^ in 1 mol·L^−1^ H_2_SO_4_ solution, and the time taken for the cell voltage to reach 10 V was considered the life of the electrode. Figure 10A shows the evolution of cell voltage with electrolysis time for Ti/Ta-Ti/IrO_2_-Ta_2_O_5_ and Ti/IrO_2_-Ta_2_O_5_ electrodes. Both electrodes exhibit similar behavior, with a slight decrease in cell voltage in the early stage due to electrolyte penetration through the pores, cracks, and grain boundaries of the oxide coating. As the ALT continues, the cell voltage increases slowly until it rises rapidly in the last stage, indicating the failure of the electrode. It should be noted that there is a small step-rise of voltage during the electrolysis for the electrode with the interlayer, which is related to the replacement of the electrolyte. The ALT life of the Ti/Ta-Ti/IrO_2_-Ta_2_O_5_ electrode was found to be 779 ± 18 h, more than twice that of the Ti/IrO_2_-Ta_2_O_5_ electrode (348 ± 21 h). Additionally, the initial cell voltage of the Ti/Ta-Ti/IrO_2_-Ta_2_O_5_ electrode is lower, indicating better electrochemical activity than the Ti/IrO_2_-Ta_2_O_5_ electrode. These results demonstrate that the addition of a Ta-Ti interlayer via DGPSAT significantly improves the service life of the electrode, which is attributed to the high corrosion resistance of the Ta-Ti interlayer, providing effective protection for the Ti substrate [24,28,29]. 

To demonstrate the corrosion resistance of the Ta-Ti interlayer and the Ti substrate, potentiodynamic polarization curves for the Ta-Ti interlayer and Ti substrate in 1 mol L^−1^ H_2_SO_4_ solution were measured, as shown in Figure 10B. The results indicate that both the Ti substrate and the Ta-Ti alloy interlayer exhibit similar passivation behavior in sulfuric acid solution. However, the passivation current density of the Ta-Ti interlayer is approximately an order of magnitude lower than that of the Ti substrate. Additionally, the corrosion potential of the Ta-Ti alloy layer is 149 mV higher than that of titanium, and the corrosion current density of the Ta-Ti interlayer is much smaller than that of the Ti substrate. These results suggest that the corrosion resistance of the Ti substrate is greatly enhanced after Ta-Ti alloying via DGPSAT. The Ta-Ti alloy layer can protect the Ti substrate effectively, thereby enhancing electrode stability and extending electrode lifespan. 

Moreover, the surface of the Ti/Ta-Ti/IrO_2_-Ta_2_O_5_ electrode exhibits reduced micro-cracks and less agglomerates of IrO_2_ nanoparticles, which can slow the infiltration of electrolyte solution through the cracks of IrO_2_-Ta_2_O_5_ coating to the substrate and avoid the fast dissolution and detachment of IrO_2_ agglomerates precipitated on the surface. The large electrochemically active surface area of the Ti/Ta-Ti/IrO_2_-Ta_2_O_5_ electrode can also lower the real working current density during the electrolysis. All of these effects can improve the stability of the oxide electrode. 

It should be emphasized that the specific micro-mechanism for the Ta-Ti interlayer to enhance the stability of the oxide anode still needs to be studied further, though it is generally ascribed to the protection of the interlayer for the Ti substrate [24,28]. The addition of a Ta-Ti interlayer will increase the cost of the oxide electrode; however, it should be cost-effective considering the high performance of the electrode with the interlayer. Furthermore, although it has been ascertained that deposition of a Ta-Ti interlayer via DGPSAT can significantly enhance the electrochemical performance of the IrO_2_-Ta_2_O_5_ electrode, optimization of the preparation processes is still required, and the fabrication of a large-size electrode using this method is also a challenge.

## 4. Conclusions

A Ta-Ti alloy interlayer has been deposited on a Ti substrate via the double-glow plasma surface alloying technology to prepare a Ti/Ta-Ti/IrO_2_-Ta_2_O_5_ composite electrode. The microstructures of the interlayer and the oxide electrodes have been examined using SEM, EDS, XRD and XPS, and the electrochemical properties of the Ti/IrO_2_-Ta_2_O_5_ and Ti/Ta-Ti/IrO_2_-Ta_2_O_5_ electrodes have been tested via LSV, EIS, CV and ALT, respectively. The following conclusions can be drawn from this investigation: 

(1) The Ta-Ti interlayer prepared using double-glow plasma surface alloying technology presents a relatively flat and uniform morphology with agglomerated cellular particles on the surface. The interlayer has a varying composition of continuously increasing Ti content and decreasing Ta content in depth. XRD analysis revealed that the Ta-Ti alloy interlayer is composed of solid solutions with β phases. XPS analysis showed that the top-surface layer of the Ta-Ti interlayer is composed of metallic Ta and a small amount of Ta oxides, while the sub-surface consists of Ta and conductive Ta suboxides. 

(2) The Ti/Ta-Ti/IrO_2_-Ta_2_O_5_ electrode has fewer micro-cracks and less aggregation of nano-IrO_2_ particles than the Ti/IrO_2_-Ta_2_O_5_ electrode. XRD analysis showed that the Ta-Ti interlayer can reduce the grain size of rutile IrO_2_. No significant differences in surface composition were detected for the two electrodes. The Ir/Ta ratio in the superficial layer for both electrodes is smaller than that in the oxide coating due to surface segregation.

(3) The Ti/Ta-Ti/IrO_2_-Ta_2_O_5_ electrode has an obviously higher electrocatalytic activity for oxygen evolution than the Ti/IrO_2_-Ta_2_O_5_ electrode, which is attributed to the introduction of the interlayer that reduces the aggregation of IrO_2_ nanoparticles and refines the IrO_2_ crystallites, thereby increasing the electrochemically active surface area. The accelerated life test demonstrates that the Ti/Ta-Ti/IrO_2_-Ta_2_O_5_ electrode has a much higher stability than the Ti/IrO_2_-Ta_2_O_5_ electrode, which is attributed to the high corrosion resistance of the Ta-Ti alloy interlayer and the effective protection of the Ti substrate. Furthermore, the reduced surface micro-cracks, fewer agglomerates of IrO_2_ particles, and the enlargement of the electrochemically active surface area via the introduction of the interlayer also improve the stability of the oxide electrode.

## Figures and Tables

**Table 1:** The processing parameters for the preparation of Ta-Ti alloy interlayer via DGPSAT.

Items	Parameters
Working temperature (°C)	900
Deposition time (h)	2.5
Working pressure (Pa)	35
Cathode (workpiece) voltage (V)	400–450
Cathode power (W)	700–750
Source voltage (V)	900–950
Source power (W)	1100–1200
Chamber gas	Ar_2_
Gas flow rates (SCCM)	35

**Table 2:** The element contents (atom %) of different samples detected by EDS.

Sample	Ti	Ta	O	Ir	Cl
Ta-Ti interlayer	1.59	92.83	5.58	–	–
Ti/IrO_2_-Ta_2_O_5_	8.12	24.34	12.09	54.24	1.21
Ti/Ta-Ti/IrO_2_-Ta_2_O_5_	2.12	31.16	12.39	53.19	1.14

**Table 3:** The element contents (atom %) of different samples detected using XPS.

Sample	Ta	O	Ir
Ti/Ta-Ti interlayer before sputtering	75.1	24.9	–
Ti/Ta-Ti interlayer after sputtering	94.1	5.9	–
Ti/IrO_2_-Ta_2_O_5_	13.54	73	13.46
Ti/Ta-Ti/IrO_2_-Ta_2_O_5_	15.75	71.91	12.34

**Table 4:** Parameters derived from EIS simulation for Ti/IrO_2_-Ta_2_O_5_ and Ti/Ta-Ti/ IrO_2_-Ta_2_O_5_ electrodes.

Electrode	*R*_s_/Ω·cm^2^	*R*_ct_/Ω·cm^2^	*Q*_dl_/Ω^−1^·cm^−2^·S^n^	*n*
Ti/IrO_2_-Ta_2_O_5_	8.1 ± 0.1	22.42 ± 1.54	0.013 ± 0.004	0.92 ± 0.01
Ti/Ta-Ti/IrO_2_-Ta_2_O_5_	7.8 ± 0.1	5.26 ± 0.81	0.031 ± 0.003	0.91 ± 0.01

**Table 5:** The double-layer capacitance and various voltametric charges of the Ti/IrO_2_-Ta_2_O_5_ and Ti/Ta-Ta/IrO_2_-Ta_2_O_5_ electrodes derived from CV measurements.

Electrode	*C*_dl_/mF·cm^−2^	$q_{tot}^{*}$/mC·cm^−2^	$q_{out}^{*}$/mC·cm^−2^	$q_{in}^{*}\;$
Ti/Ta-Ti/IrO_2_-Ta_2_O_5_	23.1 ± 0.9	175.4 ± 5.7	22.5 ± 1.2	152.9 ± 4.8
Ti/IrO_2_-Ta_2_O_5_	19.0 ± 0.5	123.2 ± 4.3	19.5 ± 0.8	103.7 ± 3.7

## Data Availability

The data that support the results of this study are available from the corresponding author upon reasonable request.
